# Supplementary material for: Ionic Organic Solid 1,3-Bis(sulfomethyl)imidazoliumate as an Effective Metal-Free Catalyst for Sustainable Organic Syntheses
Source: Molecules. 2023 Mar 16;28(6):2695. doi: 10.3390/molecules28062695 (PMC10057919; doi:10.3390/molecules28062695)
Supplement: Supplementary file 1 [file molecules-28-02695-s001.zip › molecules-2273566-supplementary.pdf]

molecules

*Supplementary Information*

# Ionic Organic Solid 1,3-bis(Sulfomethyl)Imidazoliumate As an Effective Metal-Free Catalyst for Sustainable Organic Syntheses

**Mario Martos, Angélica M. Guapacha and Isidro M. Pastor ***

Organic Chemistry Department and Institute of Organic Synthesis (ISO), University of Alicante, ctra. San Vicente del Raspeig s/n, 03690, San Vicente del Raspeig, Alicante, Spain
* Correspondence: ipastor@ua.es; Tel.: +34-965903728

## Contents

**Detailed Experimental Protocols** .......... 2
**Spectral data for all compounds** .......... 3
**NMR spectra** .......... 6
**References** .......... 20

## Detailed Experimental Protocols

### Procedure for the preparation of bsmim

Glyoxal (40% aq., 5 mmol, 0.57 mL), formaldehyde (37% aq., 5 mmol, 0.37 mL) and aminomethanesulfonic acid (10 mmol, 1.11 g) were added to a round-bottom flask along with water (2 mL) and sulfuric acid (catalytic, 100 µL) and stirred at 95 ºC for 20 minutes, after which it was quickly put in an ice-brine bath at -15 ºC. Then, acetone (20 mL) was added and the mixture was vigorously stirred (1200 rpm), which caused the precipitation of a fluffy brown solid over the next 20 minutes. The solid was then filtered, affording 628 mg of pure **bsmim** (49% yield).

### General procedure for the Friedländer synthesis of quinolines promoted by bsmim

2-Aminobenzophenone (0.5 mmol), ketone (2.5 mmol) and **bsmim** (10 mol%, 13 mg) were added to a reaction tube. The reaction was then stirred at 80 ºC for 16 hours, after which ethyl acetate was added (1 mL). After filtering the organic phase to remove the catalyst and removing the solvent under vacuum, the crude product was purified by column chromatography using mixtures of hexane and ethyl acetate.

### General procedure for the allylation of heterocycles with bsmim

In a glass tube, precisely weighed allyl alcohol (0.5 mmol, 105 mg), heterocycle (0.5 mmol) and **bsmim** (10 mol%, 13 mg) were added. The mixture was then stirred at 80 ºC until completion (monitored by GC-MS), after which the crude reaction mixture was diluted with ethyl acetate (1 mL) and filtered through a thin plug of silica to remove the catalyst. After evaporation of the solvent under reduced pressure, the corresponding allyl heterocycles were obtained.

## Spectral data for all compounds

**1,3-Bis(sulfomethyl)imidazole (1):** Light brown solid, 49% yield; m.p. = 291-294 ºC; $^{1}H$ NMR (400 MHz, $D_2O$) $\delta_H$ = 9.26 (s, 1H, NCHN), 7.76 (d, *J* = 1.6 Hz, 2H, NCHCHN), 5.43 (s, 4H, $CH_2$); $^{13}C$ NMR (100 MHz, $D_2O$) $\delta_C$ = 138.3, 123.5, 62.6. MS (EI, 70 eV) *m*/*z* (%): 66 (5), 64 (100), 48 (40); MS/MS ($ESI^+$) [256]: 261 (11), 242 (26), 238 (20), 193 (13), 176 (100), 163 (27).

**3-Acetyl-2-methyl-4-phenylquinoline (3):** Yellow solid, purification by column chromatography (hexane/ethyl acetate 7:3), 99% yield; m.p. 120-122 ºC (lit. 117-119 ºC)[1]; $^{1}H$ NMR (300 MHz, $CDCl_3$): $\delta_H$ = 8.13 (d, *J* = 8.4 Hz, 1H, $CH_{Ar}$), 7.74 (ddd, *J* = 8.4, 6.9, 1.4 Hz, 1H, $CH_{Ar}$), 7.65-7.62 (m, 1H, $CH_{Ar}$), 7.53-7.46 (m, 4H, $CH_{Ar}$), 7.38-7.35 (m, 2H, $CH_{Ar}$), 2.73 (s, 3H, $NCCH_3$), 2.00 (s, 3H, $COCH_3$); $^{13}C$ NMR (100 MHz, $CDCl_3$): $\delta_C$ = 205.5, 153.7, 147.1, 144.7, 135.2, 134.9, 130.6, 130.1, 129.2, 128.9, 128.6, 126.9, 126.4, 125.2, 32.1, 23.7; MS (EI, 70 eV) *m*/*z* (%): 261 ($M^+$, 49), 247 (19), 246 (100), 219 (7), 218 (38), 217 (31), 176 (22), 151 (8).

**3-(Ethoxycarbonyl)-2-methyl-4-phenylquinoline (4):** Yellow solid, purification by column chromatography (hexane/ethyl acetate 9:1), 98% yield; m.p. 99-100 ºC (lit. 98-100 ºC)[1]; $^{1}H$ NMR (400 MHz, $CDCl_3$): $\delta_H$ = 8.06 (d, *J* = 8.1 Hz, 1H, $CH_{Ar}$), 7.68 (ddd, *J* = 8.4, 6.8, 1.4 Hz, 1H, $CH_{Ar}$), 7.56 (dd, *J* = 8.4, 0.9 Hz, 1H, $CH_{Ar}$), 7.47-7.43 (m, 3H, $CH_{Ar}$), 7.41-7.33 (m, 3H, $CH_{Ar}$), (q, *J* = 7.1 Hz, 2H, $CH_2$), 2.78 (s, 3H, $NCCH_3$), 0.92 (t, *J* = 7.1 Hz, 3H, $COCCH_3$); $^{13}C$ NMR (100 MHz, $CDCl_3$): $\delta_C$ = 168.5, 154.6, 147.7, 146.4, 135.8, 130.3, 129.4, 128.9, 128.5, 128.3, 127.5, 126.5, 126.5, 125.2, 61.4, 23.8, 13.7; MS (EI, 70 eV) *m*/*z* (%): 292 ($M^+$+1, 20), 291 ($M^+$, 96), 263 (8), 262 (8), 247 (20), 246 (100), 245 (34), 219 (10), 218 (44), 217 (41), 216 (14), 177 (8), 176 (23), 151 (8).

**3-acetyl-6-chloro-2-methyl-4-phenylquinoline (5):** Yellow solid, purification by column chromatography (hexane/ethyl acetate 85:15), 93% yield; m.p. 160-161 ºC (lit. 149-152 ºC)[1]; $^{1}H$ NMR (400 MHz, $CDCl_3$): $\delta_H$ = 8.06 (d, *J* = 8.9 Hz, 1H, $CH_{Ar}$), 7.66 (dd, *J* = 8.9, 2.3 Hz, 1H, $CH_{Ar}$), 7.58 (d, *J* = 2.3 Hz, 1H, $CH_{Ar}$) 7.55-7.52 (m, 3H, $CH_{Ar}$), 7.35-7.32 (m, 2H, *J* = 8.9, 2.3 Hz, 1H, $CH_{Ar}$),, 2.69 (s, 3H, $NCCH_3$), 1.99 (s, 3H, $COCH_3$); $^{13}C$ NMR (100 MHz, $CDCl_3$): $\delta_C$ = 204.9, 154.1, 145.3, 143.9, 135.7, 134.4, 132.9, 131.5, 130.0, 129.5, 129.1, 126.1, 125.1, 31.9, 23.6; MS (EI, 70 eV) *m*/*z* (%): 297 ($M^+$+2, 16), 296 ($M^+$+1, 10), 295 ($M^+$, 46), 282 (33), 281 (19), 280 (100), 254 (7), 252 (22), 218 (8), 217 (32), 216 (13), 189 (8), 176 (23).

**9-Phenyl-1,2,3,4-tetrahydroacridine (6):** Yellow solid, purification by column chromatography (hexane/ethyl acetate 8:2), 94% yield; m.p. 151-153 ºC (lit. 152-155 ºC)[2]; $^{1}H$ NMR (300 MHz, $CDCl_3$): $\delta_H$ = 8.13 (d, *J* = 8.5 Hz, 1H, $CH_{Ar}$), 7.66-7.60 (m, 1H, $CH_{Ar}$), 7.56-7.45 (m, 3H, $CH_{Ar}$), 7.35-7.33 (m, 2H, $CH_{Ar}$), 7.25-7.22 (m, 2H, $CH_{Ar}$), 3.27 (t, *J* = 6.6 Hz, 2H, $NCCH_2$), 2.62 (t, *J* = 6.4 Hz, 2H, $PhCCCH_2$), 2.02-1.93 (m, 2H, $NCCCH_2$), 1.84-1.76 (m, 2H, $PhCCCCH_2$); $^{13}C$ NMR (75 MHz, $CDCl_3$): $\delta_C$ = 158.9, 147.7, 145.4, 136.9, 129.1, 129.0, 128.8, 128.7, 128.1, 127.7, 126.9, 126.0, 125.9, 33.8, 28.1, 23.0, 22.8; MS (EI, 70 eV) *m*/*z* (%): 260 ($M^+$+1, 19), 259 ($M^+$, 100), 258 ($M^+$-1, 66), 244 (8), 230 (14), 182 (8).

**9-Phenyl-2,3-dihydro-1*H*-cyclopenta[*b*]quinoline (7):** Faint yellow solid, purification by column chromatography (hexane/ethyl acetate 98:2), 93% yield, m.p. 138-141 ºC (lit. 138-139 ºC)[2]; $^1$H NMR (400 MHz, $CDCl_3$): $\delta_H$ = 8.02-8.00 (m, 1H, $CH_{Ar}$), 7.57-7.53 (m, 2H, $CH_{Ar}$), 7.47-7.37 (m, 3H, $CH_{Ar}$), 7.32-7.28 (m, 3H, $CH_{Ar}$), 3.17 (t, *J* = 7.7 Hz, 2H, $NCCH_2$), 2.83 (t, *J* = 7.4 Hz, 2H, $PhCCCH_2$), 2.13-2.07 (m, 2H, $NCCCH_2$); $^{13}$C NMR (100 MHz, $CDCl_3$): $\delta_C$ = 167.5, 147.9, 142.9, 136.9, 133.8, 129.4, 128.9, 128.6, 128.4, 128.1, 126.4, 125.8, 125.7, 35.3, 30.5, 23.7; MS (EI, 70 eV) *m/z* (%): 246 ($M^+$+1, 18), 245 ($M^+$, 100), 244 ($M^+$-1, 80), 243 (12), 242 (12), 241 (7), 217 (9), 168 (15), 167 (10), 108 (7).

**12-Phenyl-6,7,8,9,10,11-hexahydrocycloocta[*b*]quinoline (8):** Faint yellow solid, purification by column chromatography (hexane/ethyl acetate 95:5), 96% yield; m.p. 209-211 ºC (lit. 214-215 ºC)[1]; $^1$H NMR (300 MHz, $CDCl_3$): $\delta_H$ = 8.01-7.98 (m, 1H, $CH_{Ar}$), 7.54-7.48 (m, 1H, $CH_{Ar}$), 7.46-7.36 (m, 3H, $CH_{Ar}$), 7.27-7.11 (m, 4H, $CH_{Ar}$), 3.17-3.13 (m, 2H, $NCCH_2$), 2.69-2.65 (m, 2H, $PhCCCH_2$), 1.88-1.84 (m, 2H, $CH_2$), 1.42-1.27 (m, 6H, $3xCH_2$); $^{13}$C NMR (75 MHz, $CDCl_3$): $\delta_C$ = 163.5, 146.7, 146.3, 137.7, 131.9, 129.4, 128.5, 128.4, 127.7, 127.3, 126.2, 125.5, 36.3, 31.3, 31.3, 28.2, 26.8, 25.9; MS (EI, 70 eV) *m/z* (%): 288 ($M^+$+1, 20), 287 ($M^+$, 93), 286 ($M^+$-1, 100), 272 (9), 260 (8), 259 (17), 258 (40), 256 (9), 245 (8), 244 (25), 243 (9), 242 (9), 232 (19), 231 (14), 230 (16), 217 (14), 216 (7), 202 (8), 189 (10).

**9-Phenyl-3,4-dihydroacridin-1(2*H*)-one (9):** Yellow solid, purification by column chromatography (hexane/ethyl acetate 8:2), 96% yield; m.p. 163-165 ºC (lit. 161-162 ºC)[2]; $^1$H NMR (400 MHz, $CDCl_3$): $\delta_H$ = 8.18 (d, *J* = 8.4 Hz, 1H, $CH_{Ar}$), 7.80 (ddd, *J* = 8.4, 6.6, 1.6 Hz, 1H, $CH_{Ar}$), 7.53-7.41 (m, 5H, $CH_{Ar}$), 7.19-7.17 (m, 2H, $CH_{Ar}$), 3.45 (t, *J* = 6.3 Hz, 2H, $NCCH_2$), 2.72 (t, *J* = 6.1 Hz, 2H, $COCH_2$), 2.28-2.25 (m, 2H, $COCCH_2$); $^{13}$C NMR (100 MHz, $CDCl_3$): $\delta_C$ = 197.6, 162.1, 152.5, 147.7, 137.4, 132.4, 128.4, 128.3, 128.1, 127.9, 127.7, 126.9, 124.0, 40.7, 34.1, 21.4; MS (EI, 70 eV) *m/z* (%): 274 ($M^+$+1, 19), 273 ($M^+$, 100), 272 ($M^+$-1, 94), 246 (7), 245 (47), 244 (97), 217 (32), 216 (31), 214 (8), 190 (9), 189 (16), 176 (7).

**2,9-Diphenyl-1,2,3,4-tetrahydroacridine (10):** Yellow solid, purification by column chromatography (hexane/ethyl acetate 9:1), 97% yield; $^1$H NMR (400 MHz, $CDCl_3$): $\delta_H$ = 7.98-7.96 (m, 1H, $CH_{Ar}$), 7.57-7.52 (m, 1H, $CH_{Ar}$), 7.41-7.35 (m, 3H, $CH_{Ar}$), 7.25-7.10 (m, 9H, $CH_{Ar}$), 3.37-3.22 (m, 2H, $CH_{Alk}$), 2.95 (tdd, *J* = 11.4, 4.7, 3.0 Hz, 1H, $CH_{Alk}$), 2.83 (ddd, *J* = 17.1, 4.9, 2.0 Hz, 1H, $CH_{Alk}$), 2.68 (dd, *J* = 17.1, 4.9 Hz, 1H, $CH_{Alk}$), 2.25-2.19 (m, 1H, $CH_{Alk}$), 2.13-2.02 (m, 1H, $CH_{Alk}$); $^{13}$C NMR (100 MHz, $CDCl_3$): $\delta_C$ = 158.4, 146.9, 146.6, 145.9, 136.9, 129.2, 129.0, 128.9, 128.8, 128.7, 128.7, 128.5, 127.9, 127.8, 127.0, 126.8, 126.5, 126.0, 125.7, 40.9, 36.2, 34.4, 30.3; MS (EI, 70 eV) *m/z* (%): 336 ($M^+$+1, 14), 335 ($M^+$, 65), 334 ($M^+$-1, 100), 231 (8), 230 (19), 91 (8).

***(E)*-3-(1,3-Diphenylallyl)-1*H*-indole (11):** Yellow oil, obtained pure, 99% yield; $^1$H NMR (400 MHz, $CDCl_3$): $\delta_H$ = 7.96 (br s, 1H, NH), 7.51 (d, *J* = 8.0 Hz, 1H, $CH_{Ar}$), 7.45-7.22 (m, 12H, $CH_{Ar}$), 7.10 (ddd, *J* = 8.0, 7.1, 1.0 Hz, 1H, $CH_{Ar}$), 6.93 (d, *J* = 1.3 Hz, 1H, $CH_{Ar}$), 6.80 (dd, *J* = 15.9, 7.4 Hz, 1H, PhC=CH), 6.52 (br d, *J* = 15.9 Hz, 1H, C=CHPh), 5.19 (br d, *J* = 7.4 Hz, 1H, C=CCH); $^{13}$C NMR (100 MHz, $CDCl_3$): $\delta_C$ = 143.5, 137.6, 136.8, 132.7, 130.7, 128.6, 128.6, 127.3, 126.9, 126.5, 126.5, 122.7, 122.2, 120.0, 119.6, 118.8, 111.2, 46.3; MS (EI, 70 eV) *m/z* (%): 310 ($M^+$+1, 25), 309 ($M^+$, 100), 308 ($M^+$-1, 39), 294 (10), 233 (7), 232 (36), 231 (7), 230 (20), 218 (15), 217 (17), 206 (28), 205 (8), 204 (22), 202 (8), 192 (14), 191 (16), 178 (9), 130 (19), 115 (17).

**(*E*)-3-(1,3-Diphenylallyl)-2-phenyl-1*H*-indole (12):** Faint orange oil, purified by preparative TLC (hexane/ethyl acetate 9:1), 60% yield; $^{1}$H NMR (300 MHz, $CDCl_3$): $\delta_H$ = 8.02 (br s, 1H, NH), 7.52-7.48 (m, 2H, $CH_{Ar}$), 7.44-7.12 (m, 16H, $CH_{Ar}$), 6.98 (ddd, *J* = 8.0, 7.1, 1.0 Hz, 1H, $CH_{Ar}$), 6.88 (dd, *J* = 15.8, 7.3 Hz, 1H, PhC=CH), 6.39 (dd, *J* = 15.8, 1.0 Hz, 1H, C=CHPh), 5.27 (br d, *J* = 7.3 Hz, 1H, C=CCH); $^{13}$C NMR (75 MHz, $CDCl_3$): $\delta_C$ = 143.6, 137.6, 136.3, 135.7, 133.0, 132.4, 131.2, 128.9, 128.7, 128.6, 128.4, 128.2, 128.0, 127.2, 126.4, 126.2, 122.2, 121.3, 119.8, 113.9, 111.1, 45.2; MS (EI, 70 eV) *m*/*z* (%): 386 ($M^+$+1, 30), 385 ($M^+$, 100), 384 ($M^+$-1, 19), 309 (12), 308 (46), 307 (7), 306 (17), 304 (11), 295 (21), 294 (96), 293 (29), 292 (9), 291 (11), 282 (17), 281 (8), 280 (18), 278 (8), 230 (14), 218 (9), 217 (13), 207 (9), 206 (47), 205 (8), 204 (23), 203 (8), 202 (9), 194 (9), 193 (45), 192 (22), 191 (22), 189 (8), 178 (9), 176 (7), 165 (14), 153 (9), 152 (10), 146 (9), 115 (9), 91 (7).

**(*E*)-3-(1,3-Diphenylallyl)-9-ethyl-1*H*-indole (13):** Reddish oil, obtained pure, 99% yield; $^{1}$H NMR (300 MHz, $CDCl_3$) $\delta_H$ = 7.89 (br s, 1H, NH), 7.36-7.15 (m, 11H, $CH_{Ar}$), 7.02-6.97 (m, 2H, CHN, $CH_{Ar}$), 6.84 (dd, *J* = 2.4, 0.9 Hz, 1H, $CH_{Ar}$), 6.72 (dd, *J* = 15.8, 7.4 Hz, 1H, PhC=CH), 6.43 (br d, *J* = 15.8 Hz, 1H, C=CHPh), 5.10 (br d, *J* = 7.4 Hz, 1H, C=CCH), 2.82 (q, *J* = 7.6 Hz, 2H, $CH_2$), 1.34 (t, *J* = 7.6 Hz, 3H, $CH_3$); $^{13}$C NMR (75 MHz, $CDCl_3$): $\delta_C$ = 143.6, 137.6, 135.6, 132.7, 130.6, 128.6, 128.6, 128.5, 127.3, 126.7, 126.6, 126.4, 122.3, 120.7, 119.8, 119.2, 117.8, 46.4, 24.1, 13.9; MS (EI, 70 eV) *m*/*z* (%): 338 ($M^+$+1, 27), 337 ($M^+$, 100), 336 ($M^+$-1, 34), 334 (9), 333 (28), 332 (9), 308 (20), 261 (7), 260 (33), 234 (23), 231 (9), 230 (24), 218 (9), 217 (14), 204 (10), 192 (17), 191 (20), 189 (7), 158 (15), 130 (12), 115 (11); HRMS (QTOF) calculated for $C_{25}H_{23}N$ = 337.1830, observed = 337.1823.

**(*E*)-1-(1,3-Diphenylallyl)-1*H*-pyrazole (14):** Colorless oil, obtained pure, 99% yield; $^{1}$H NMR (300 MHz, $CDCl_3$): $\delta_H$ = 7.60 (d, *J* = 1.4 Hz, 1H, $CH_{Ar}$), 7.45 (d, *J* = 2.2 Hz, 1H, $CH_{Ar}$), 7.39-7.20 (m, 10H, $CH_{Ar}$), 6.71 (dd, *J* = 16.0, 6.9 Hz, 1H, PhC=CH), 6.43 (br d, *J* = 16.0 Hz, 1H, C=CHPh), 6.30-6.28 (m, 1H, $CH_{Ar}$), 6.18 (br d, *J* = 6.9 Hz, 1H, C=CCH); $^{13}$C NMR (75 MHz, $CDCl_3$): $\delta_C$ = 139.6, 139.4, 136.1, 133.8, 128.9, 128.7, 128.2, 127.4, 127.3, 126.8, 105.7, 67.6; MS (EI, 70 eV) *m*/*z* (%): 261 ($M^+$+1, 15), 260 ($M^+$, 78), 259 ($M^+$-1, 24), 232 (7), 193 (28), 192 (43), 191 (55), 190 (11), 189 (23), 184 (8), 183 (55), 179 (7), 178 (33), 169 (31), 165 (21), 157 (18), 156 (36), 144 (21), 143 (8), 117 (9), 115 (100), 91 (27), 89 (7), 77 (8).

**(*E*)-1-(1,3-Diphenylallyl)-3,5-dimethyl-1*H*-pyrazole (15):** White solid, obtained pure, 99% yield; m.p. 108-110 ºC; $^{1}$H NMR (400 MHz, $CDCl_3$): $\delta_H$ = 7.43-7.41 (m, 2H, $CH_{Ar}$), 7.33-7.23 (m, 6H, $CH_{Ar}$), 7.16-7.14 (m, 2H, $CH_{Ar}$), 6.88 (dd, *J* = 15.9, 7.5 Hz, 1H, PhC=CH), 6.51 (br d, *J* = 15.9 Hz, 1H, C=CHPh), 5.98 (br d, *J* = 7.5 Hz, 1H, C=CCH), 5.87 (s, 1H, MeCCH), 2.27 (s, 3H, $CH_3$), 2.17 (s, 3H, $CH_3$); $^{13}$C NMR (100 MHz, $CDCl_3$): $\delta_C$ = 147.8, 140.2, 139.2, 136.5, 133.1, 128.8, 128.6, 128.0, 127.9, 127.7, 126.9, 126.9, 105.9, 64.5, 13.8, 11.5; MS (EI, 70 eV) *m*/*z* (%): 289 ($M^+$+1, 10), 288 ($M^+$, 50), 287 ($M^+$-1, 18), 273 (8), 212 (16), 211 (100), 194 (7), 193 (38), 192 (14), 185 (9), 184 (9), 178 (30), 170 (12), 165 (3), 116 (10), 115 (91), 108 (8), 91 (25).

## NMR spectra

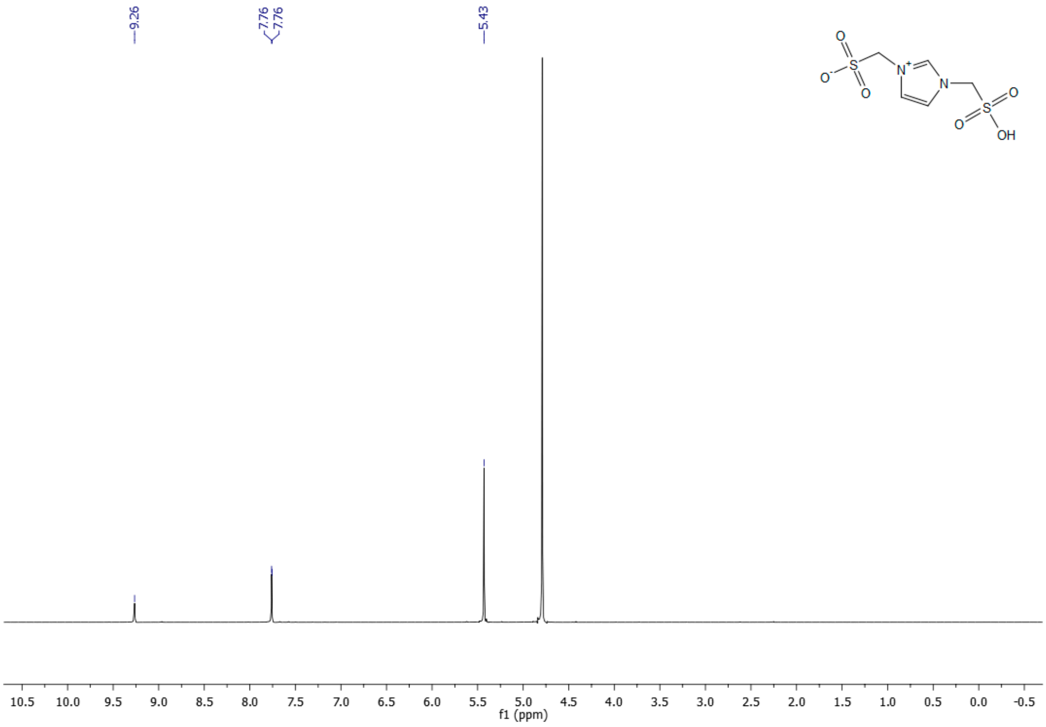


**Figure SI.1.** $^{1}H$ NMR spectrum of 1,3-bis(sulfomethyl)imidazole (**1**)

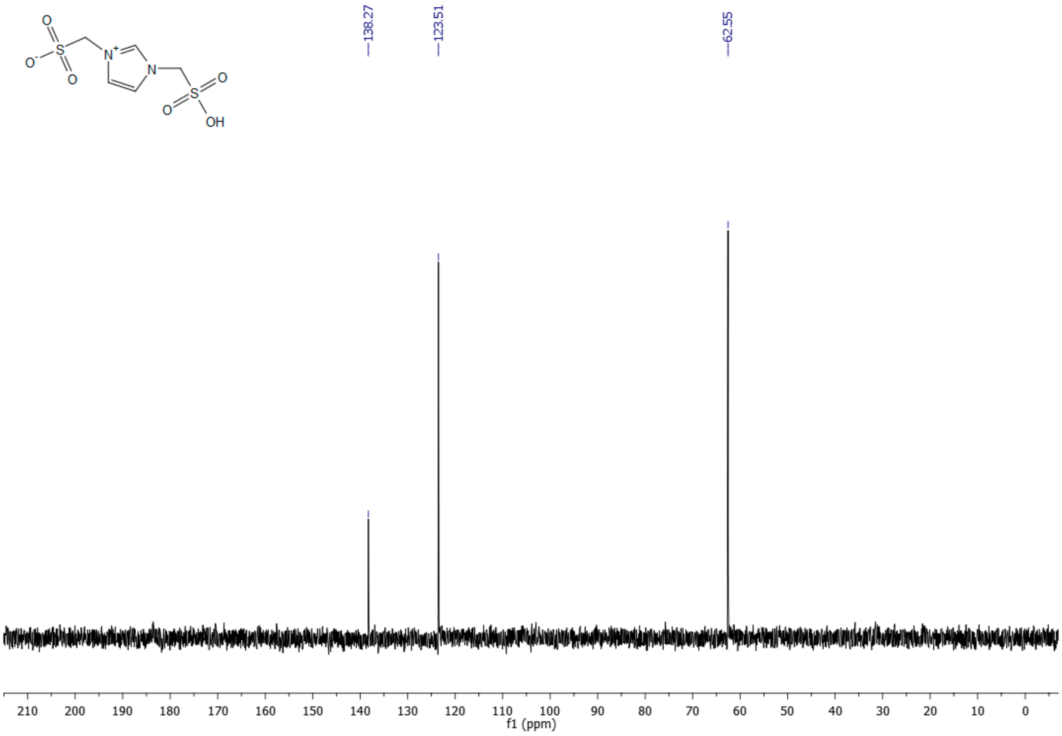


**Figure SI.2.** $^{13}C$ NMR spectrum of 1,3-bis(sulfomethyl)imidazole (**1**)

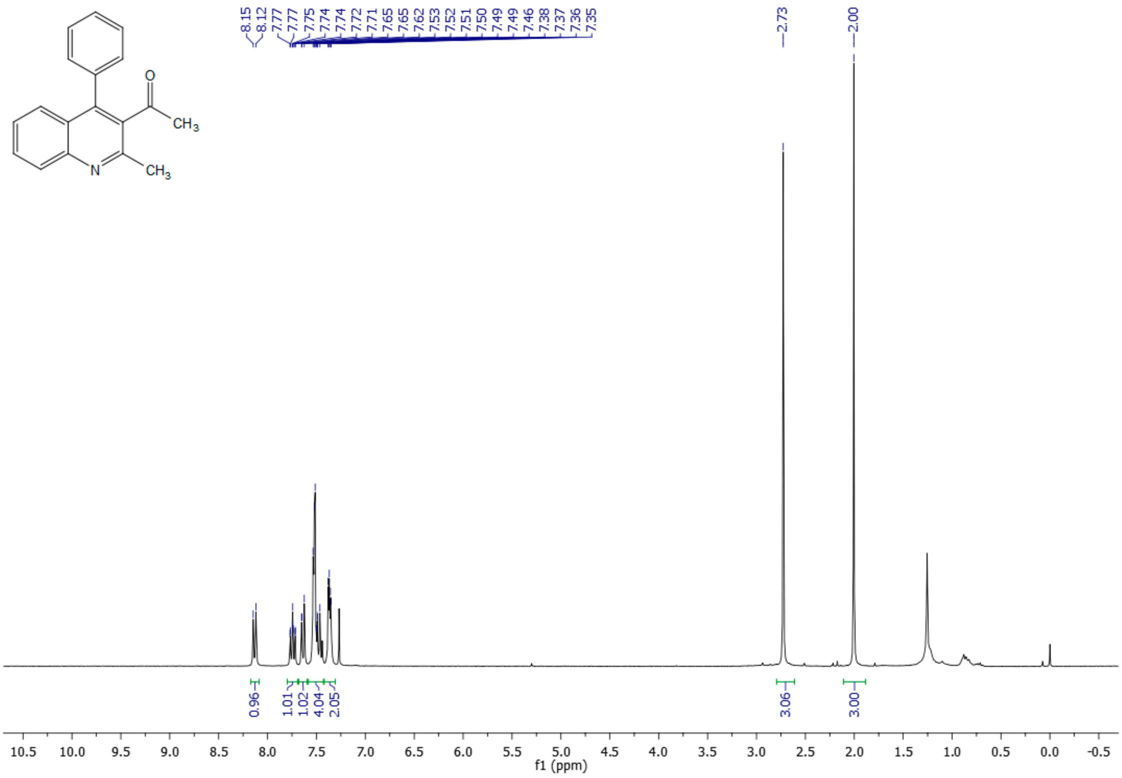


**Figure SI.3.** $^{1}H$ NMR spectrum of 3-acetyl-2-methyl-4-phenylquinoline (**3**)

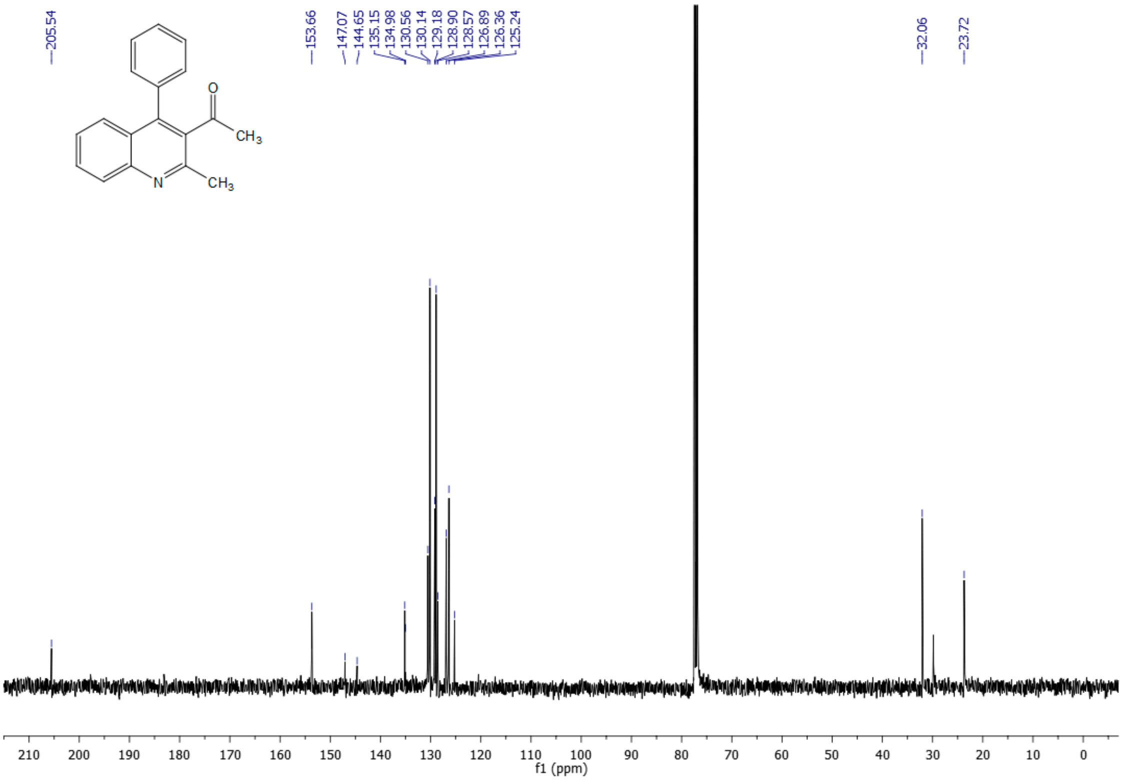


**Figure SI.4.** $^{13}C$ NMR spectrum of 3-acetyl-2-methyl-4-phenylquinoline (**3**)

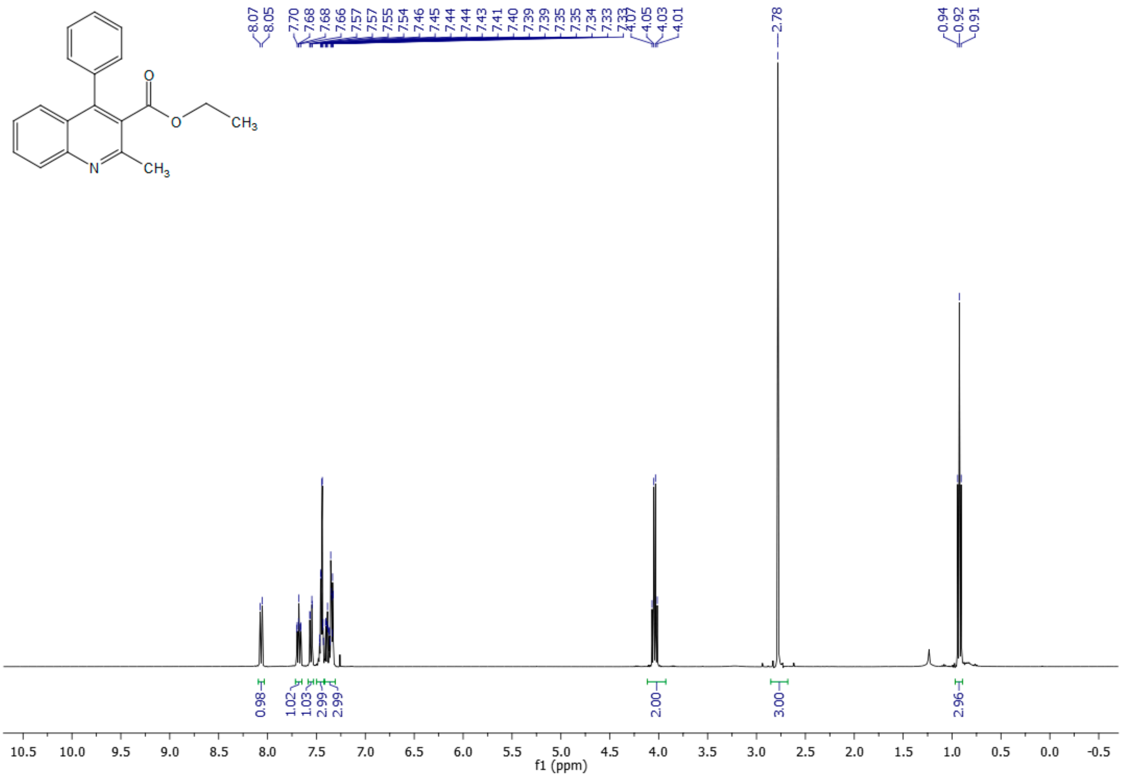

**Figure SI.5.** $^{1}H$ NMR spectrum of 3-(ethoxycarbonyl)-2-methyl-4-phenylquinoline (**4**)

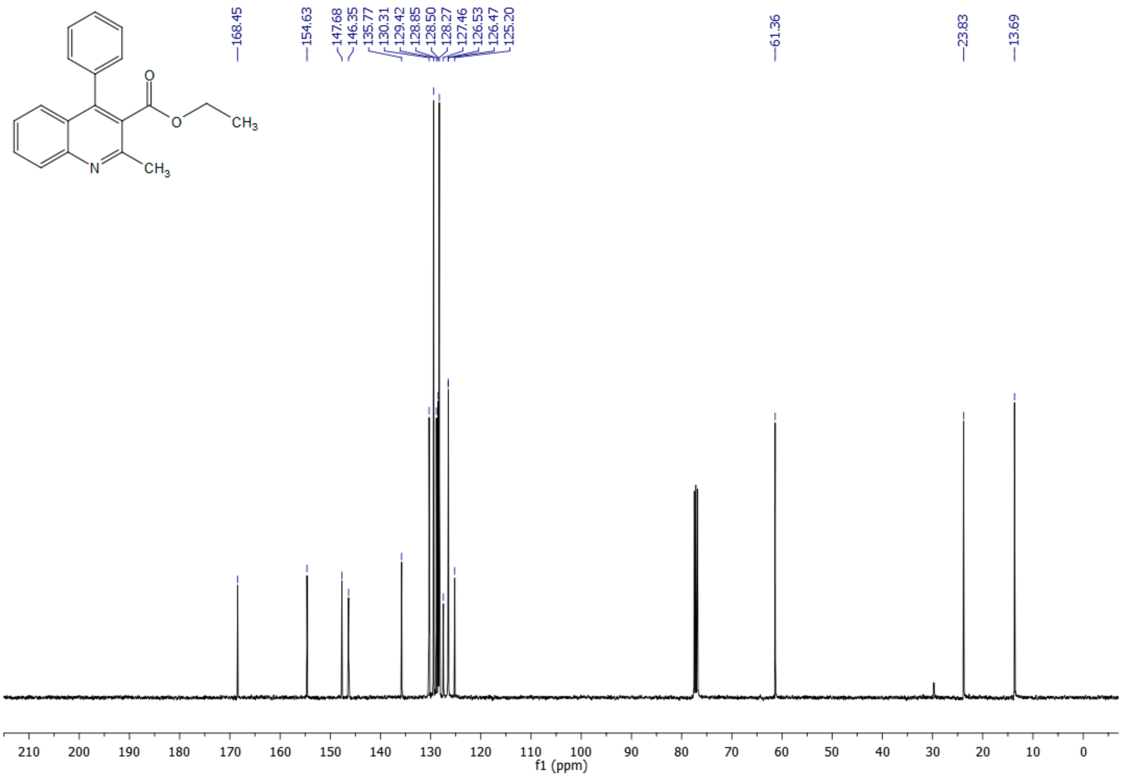

**Figure SI.6.** $^{13}C$ NMR spectrum of 3-(ethoxycarbonyl)-2-methyl-4-phenylquinoline (**4**)

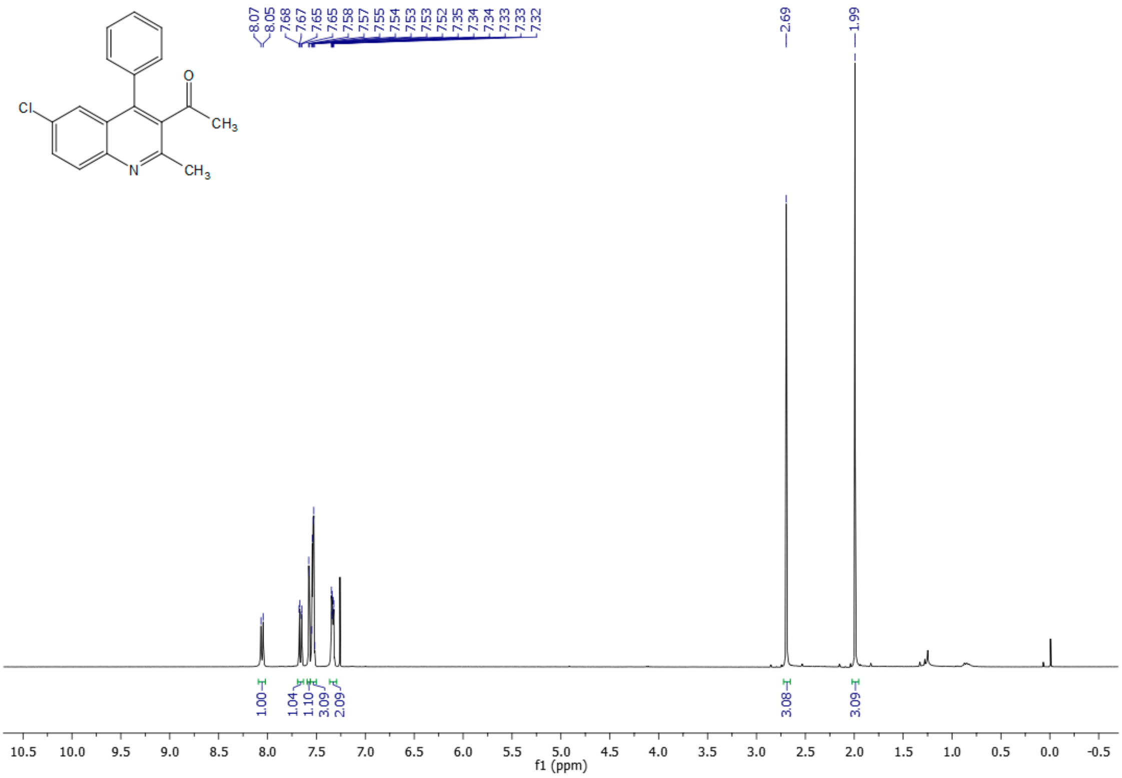


**Figure SI.7.** $^{1}$H NMR spectrum of 3-acetyl-6-chloro-2-methyl-4-phenylquinoline (**5**)

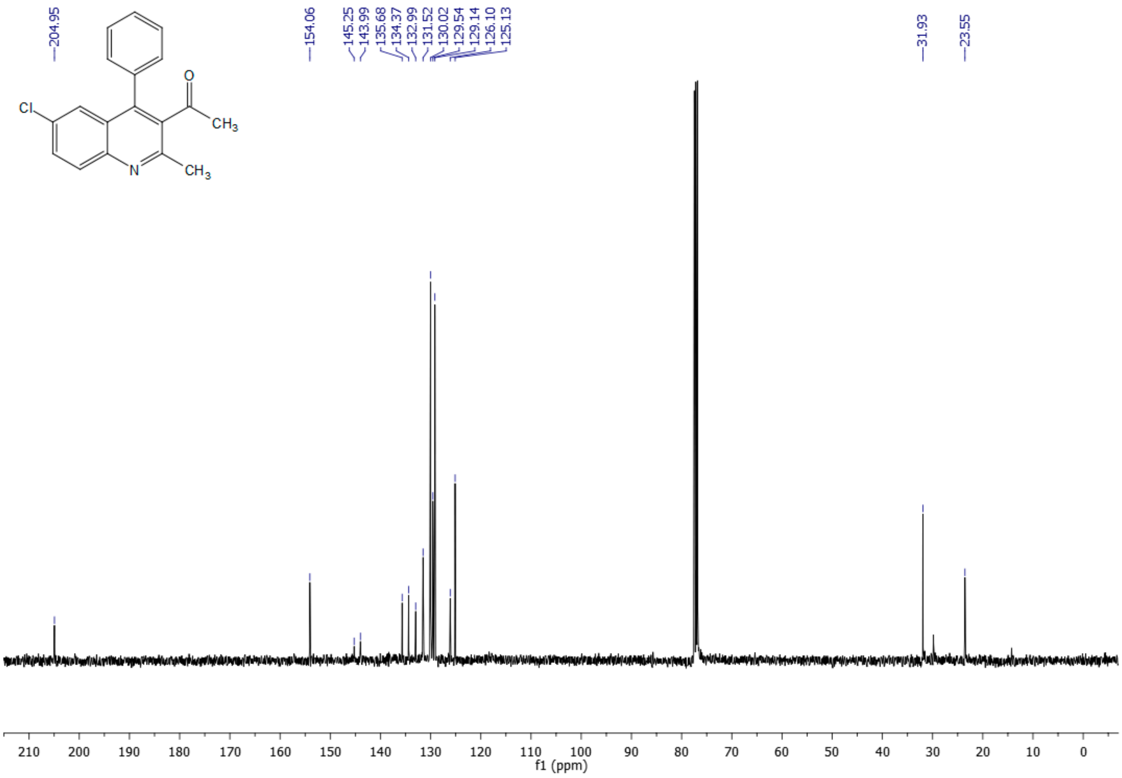


**Figure SI.8.** $^{13}$C NMR spectrum of 3-acetyl-6-chloro-2-methyl-4-phenylquinoline (**5**)

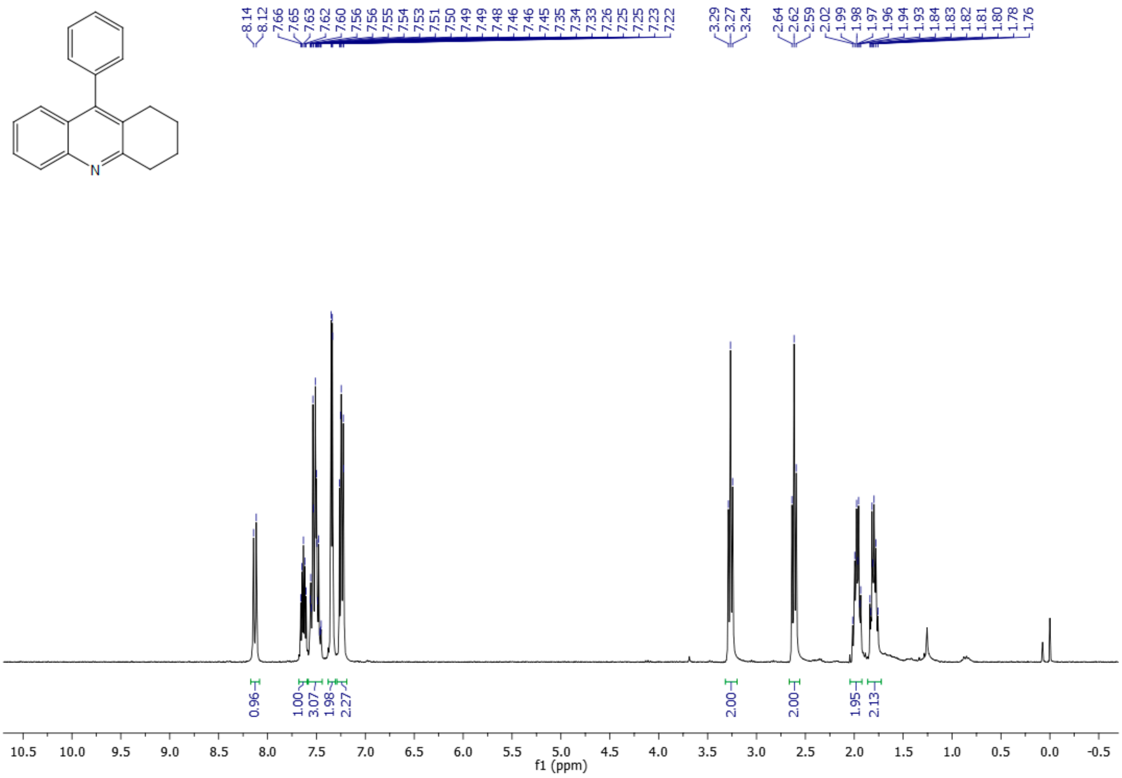

**Figure SI.9.** $^{1}H$ NMR spectrum of 9-phenyl-1,2,3,4-tetrahydroacridine (**6**)

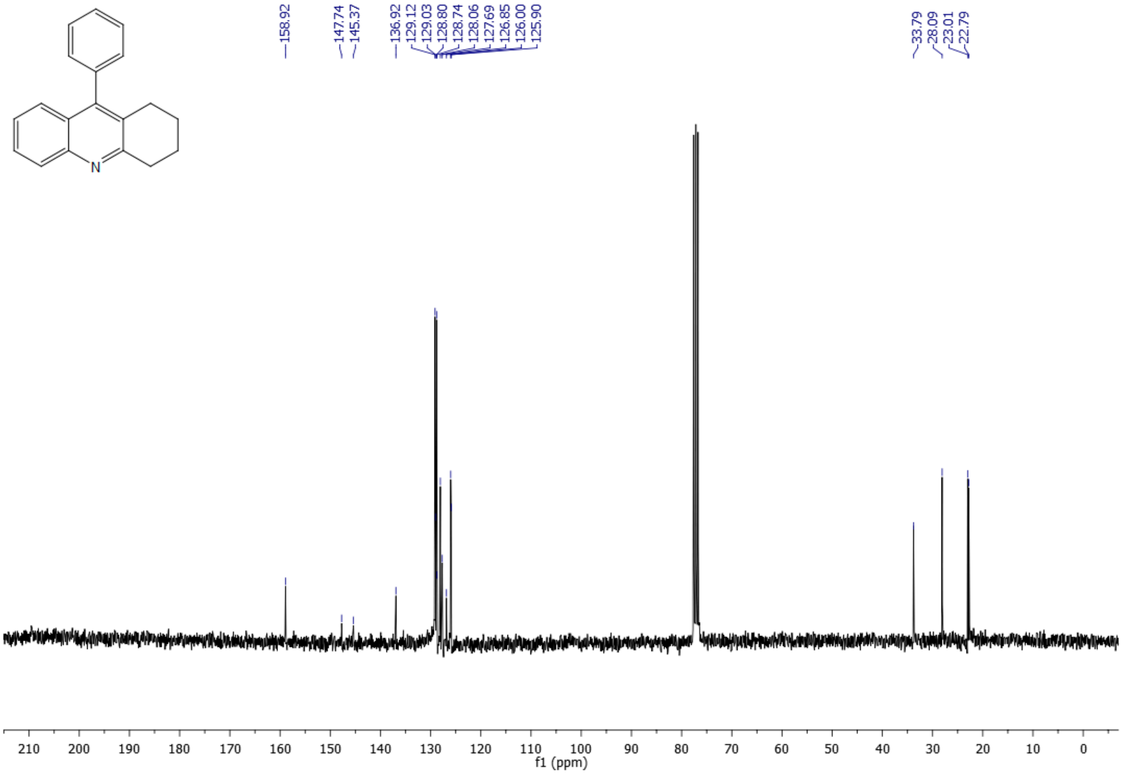

**Figure SI.10.** $^{13}C$ NMR spectrum of 9-phenyl-1,2,3,4-tetrahydroacridine (**6**)

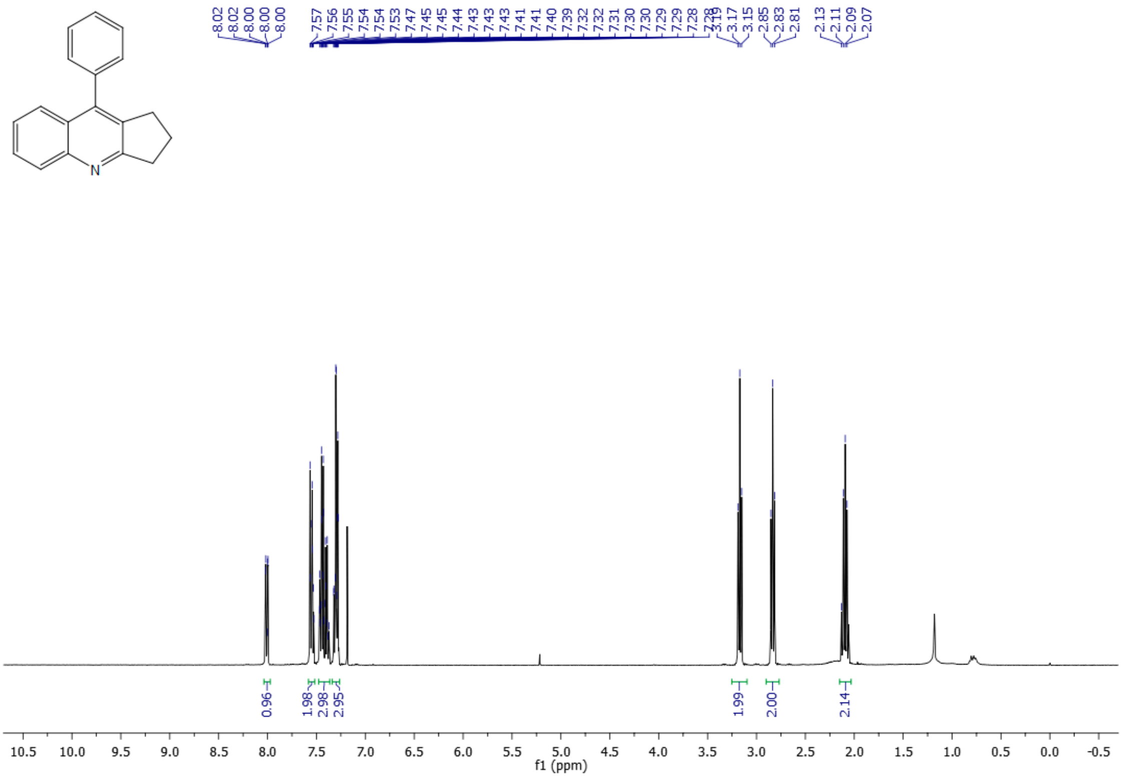


**Figure SI.11.** $^1$H NMR spectrum of 9-phenyl-2,3-dihydro-1*H*-cyclopenta[*b*]quinoline (**7**)

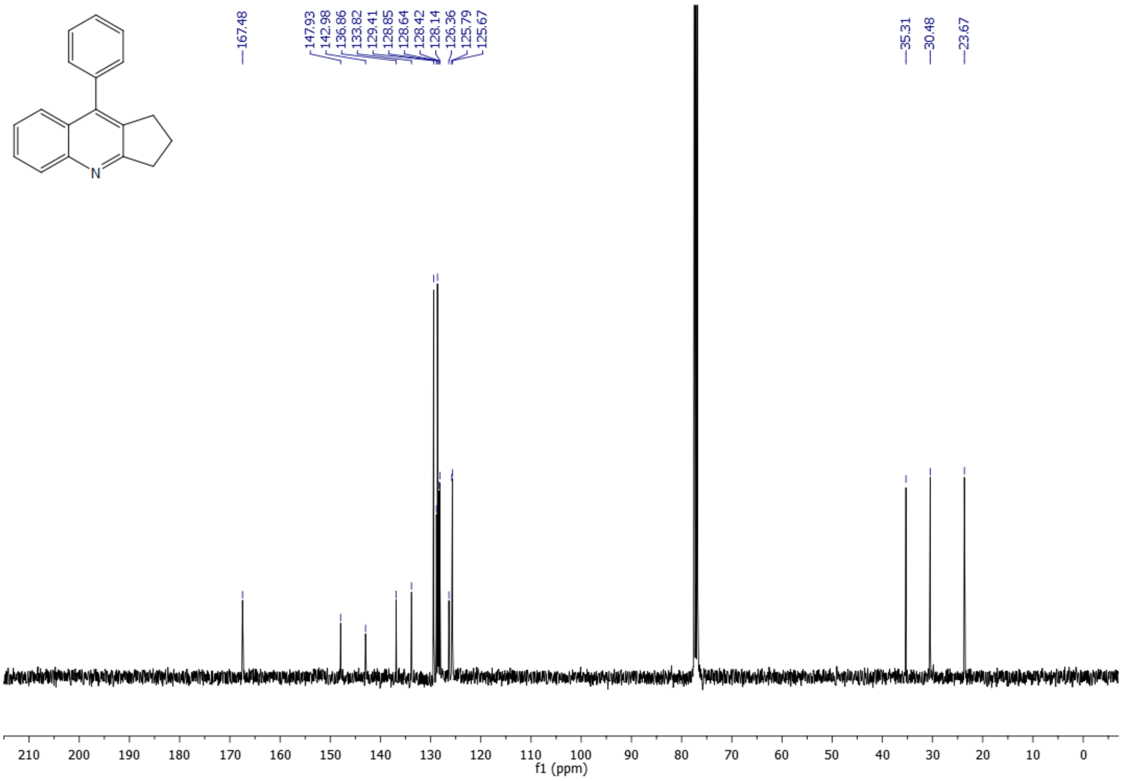


**Figure SI.12.** $^{13}$C NMR spectrum of 9-phenyl-2,3-dihydro-1*H*-cyclopenta[*b*]quinoline (**7**)

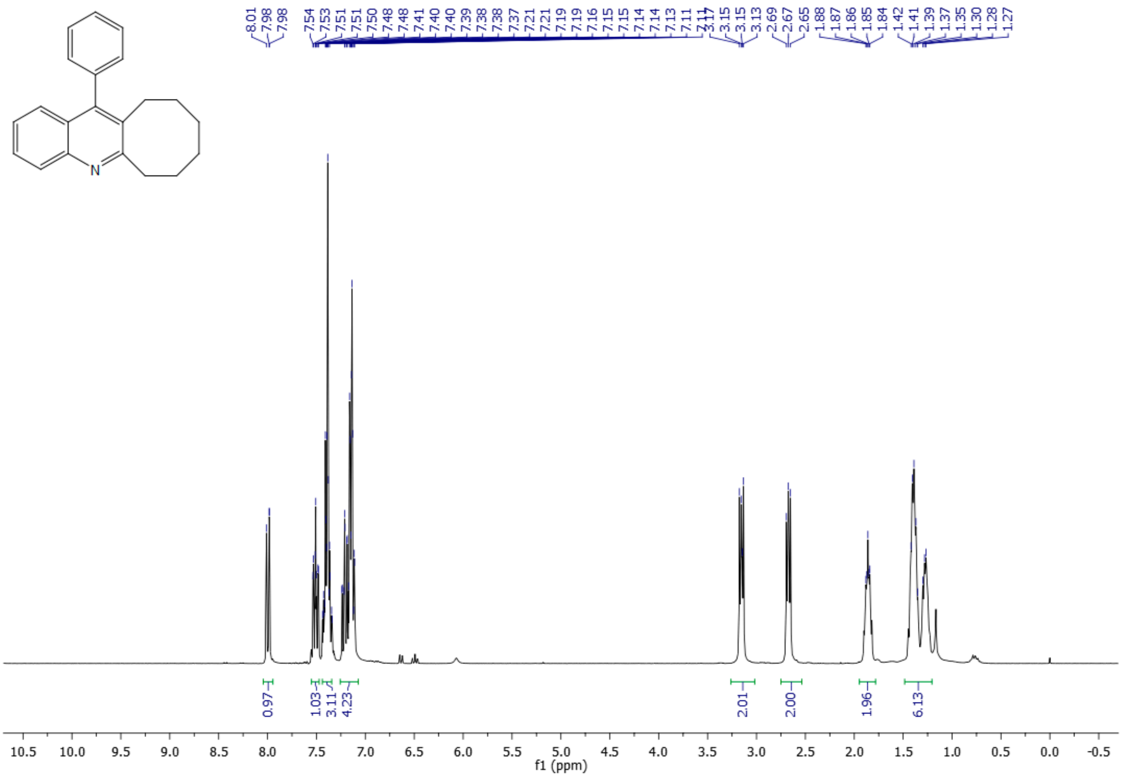


**Figure SI.13.** $^{1}$H NMR spectrum of 12-phenyl-6,7,8,9,10,11-hexahydrocycloocta[*b*]quinoline (**8**)

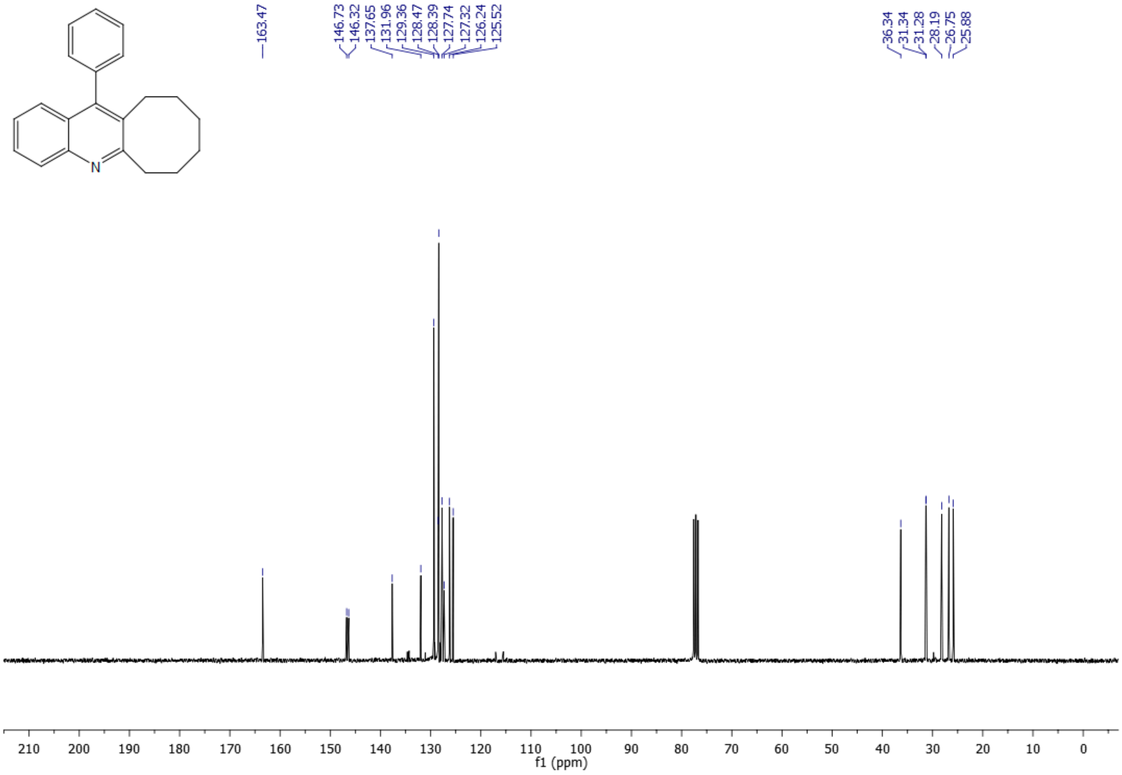


**Figure SI.14.** $^{13}$C NMR spectrum of 12-phenyl-6,7,8,9,10,11-hexahydrocycloocta[*b*]quinoline (**8**)

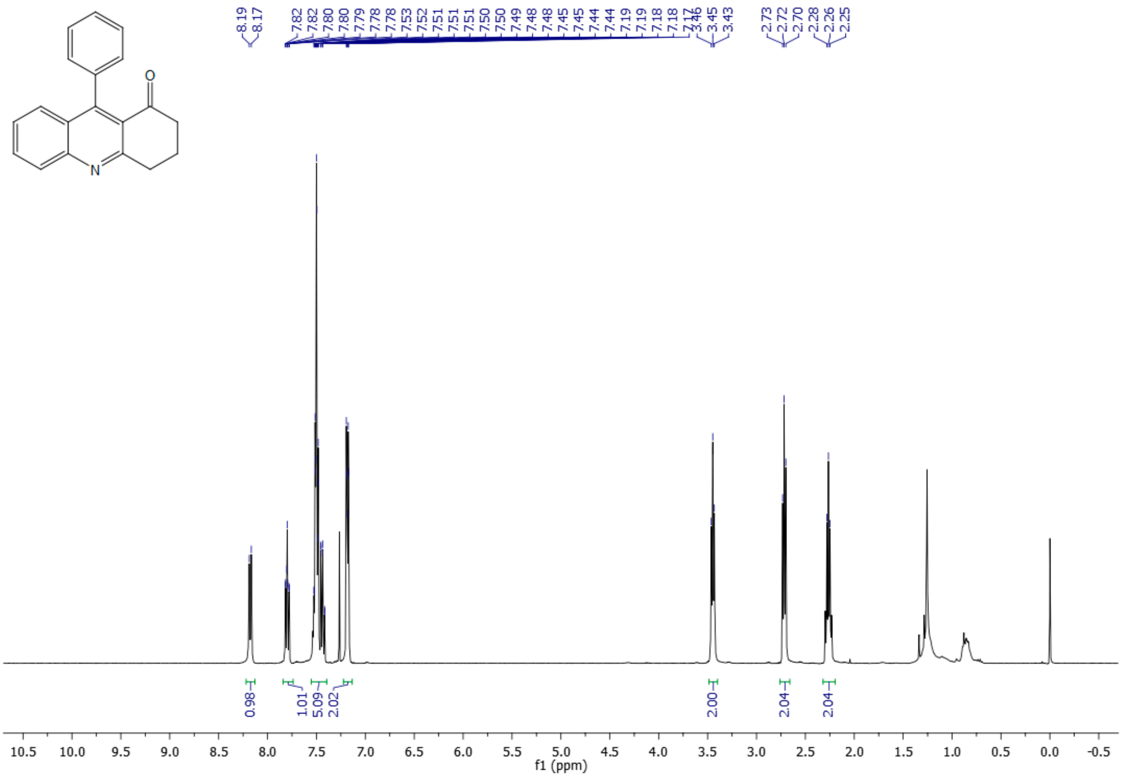


**Figure SI.15.** $^{1}$H NMR spectrum of 9-phenyl-3,4-dihydroacridin-1(2*H*)-one (**9**)

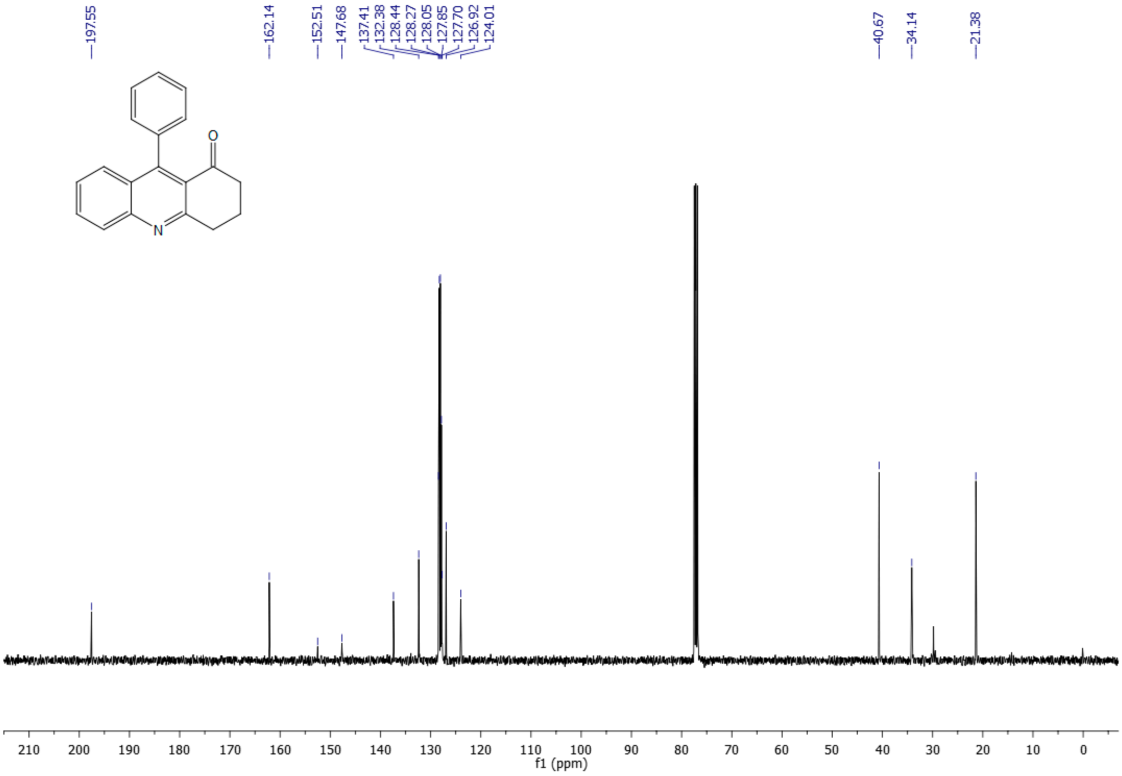


**Figure SI.16.** $^{13}$C NMR spectrum of 9-phenyl-3,4-dihydroacridin-1(2*H*)-one (**9**)

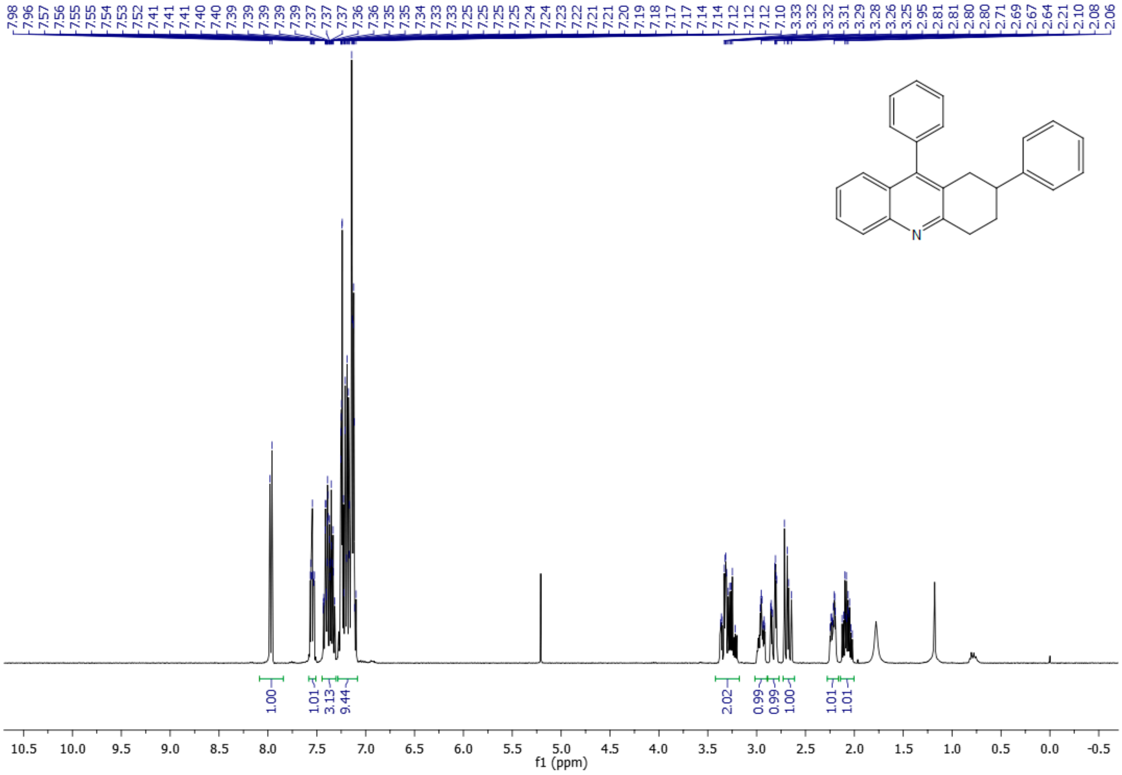

**Figure SI.17.** $^{1}H$ NMR spectrum of 2,9-diphenyl-1,2,3,4-tetrahydroacridine (**10**)

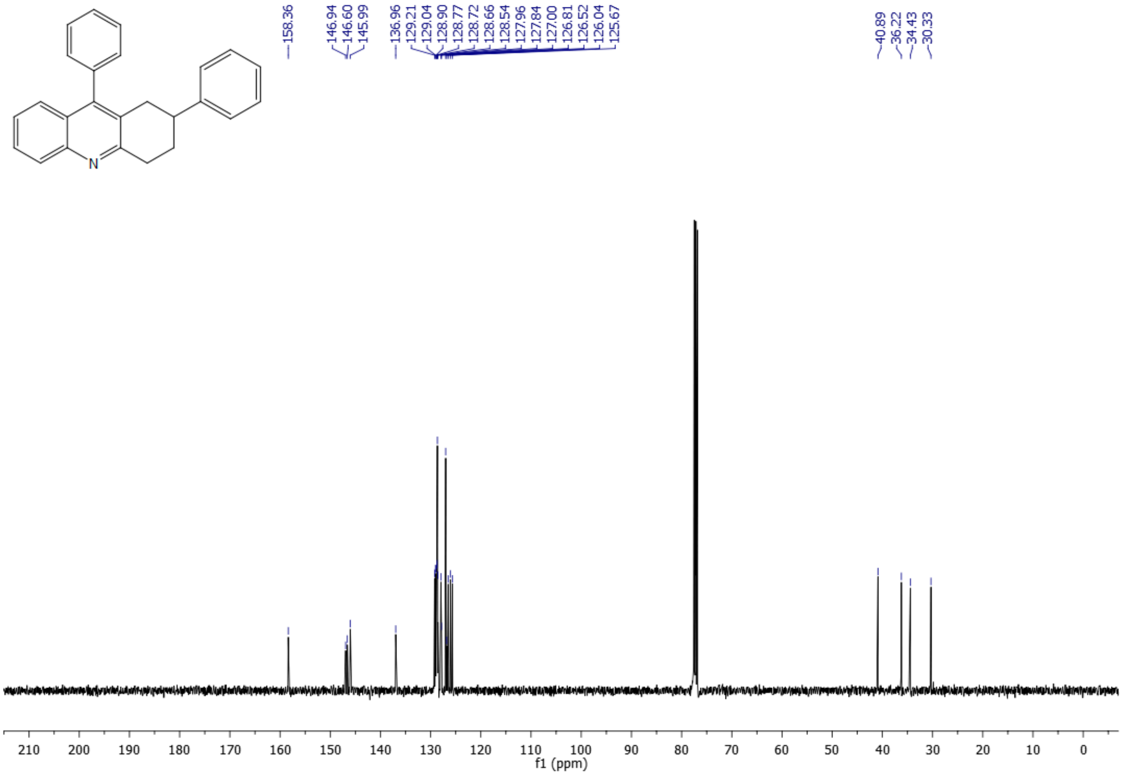

**Figure SI.18.** $^{13}C$ NMR spectrum of 2,9-diphenyl-1,2,3,4-tetrahydroacridine (**10**)

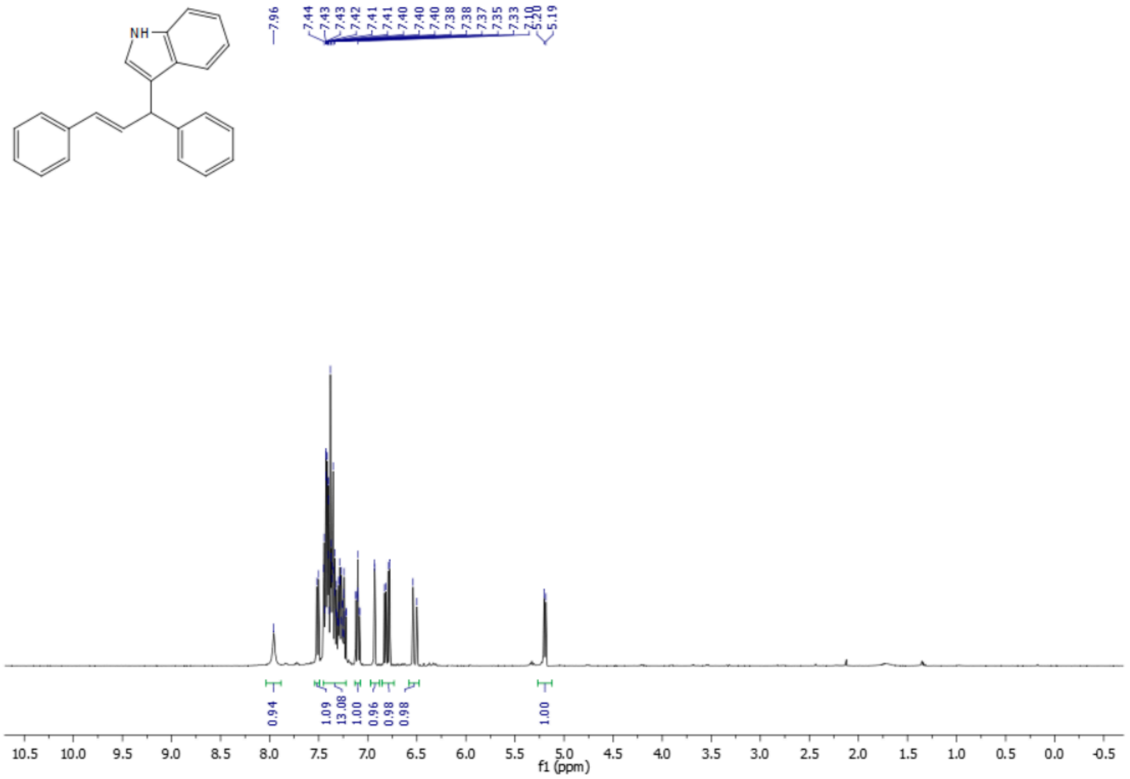


**Figure SI.19.** $^{1}H$ NMR spectrum of *(E)*-3-(1,3-diphenylallyl)-1*H*-indole (**11**)

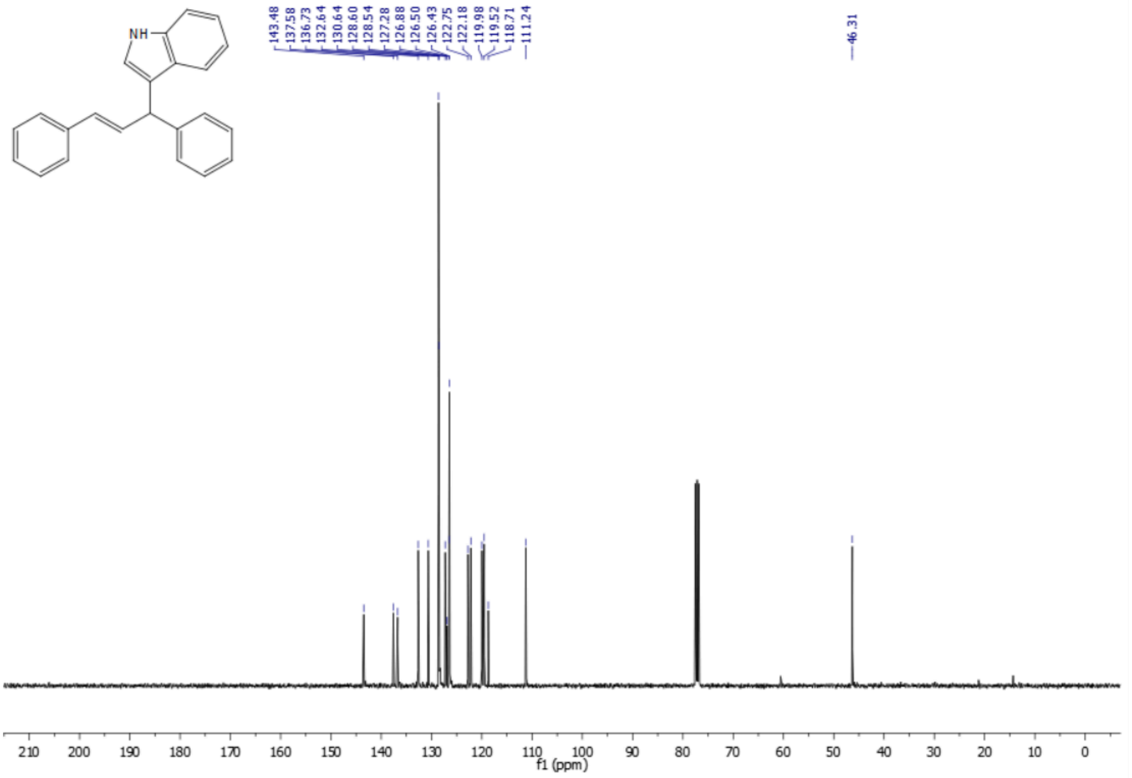


**Figure SI.20.** $^{13}C$ NMR spectrum of *(E)*-3-(1,3-diphenylallyl)-1*H*-indole (**11**)

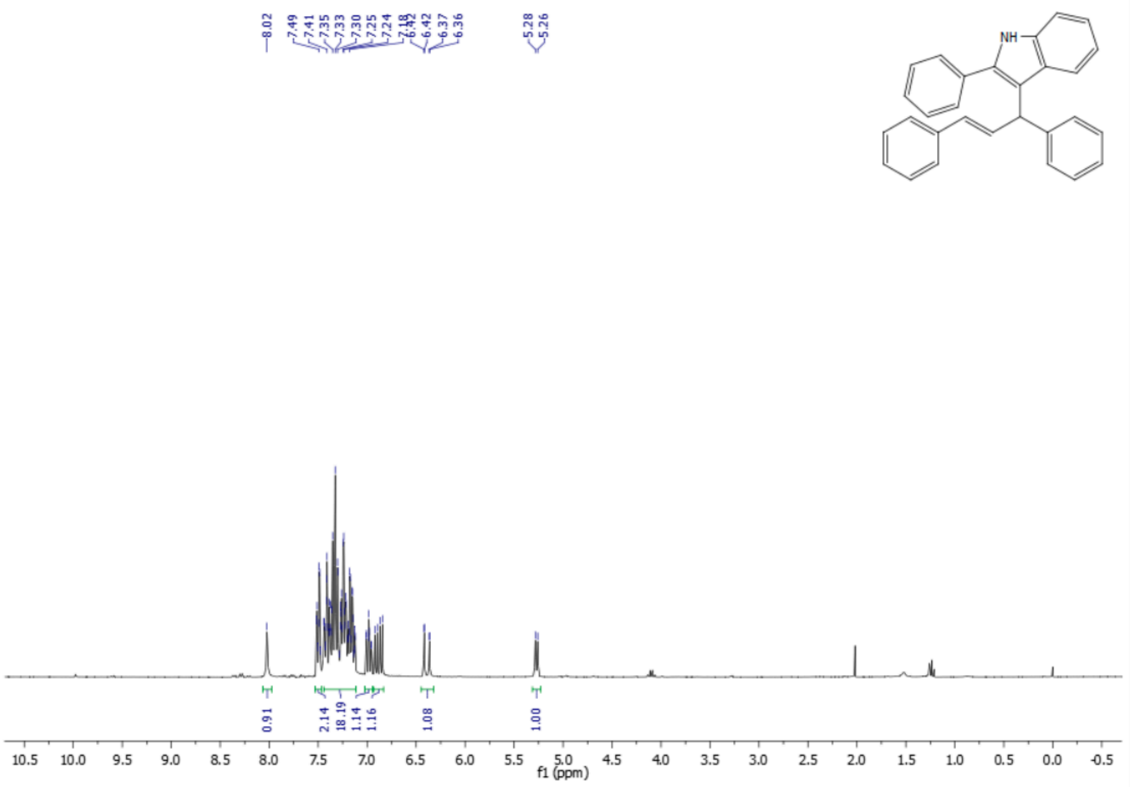


**Figure SI.21.** $^{1}$H NMR spectrum of *(E)*-3-(1,3-diphenylallyl)-2-phenyl-1*H*-indole (**12**)

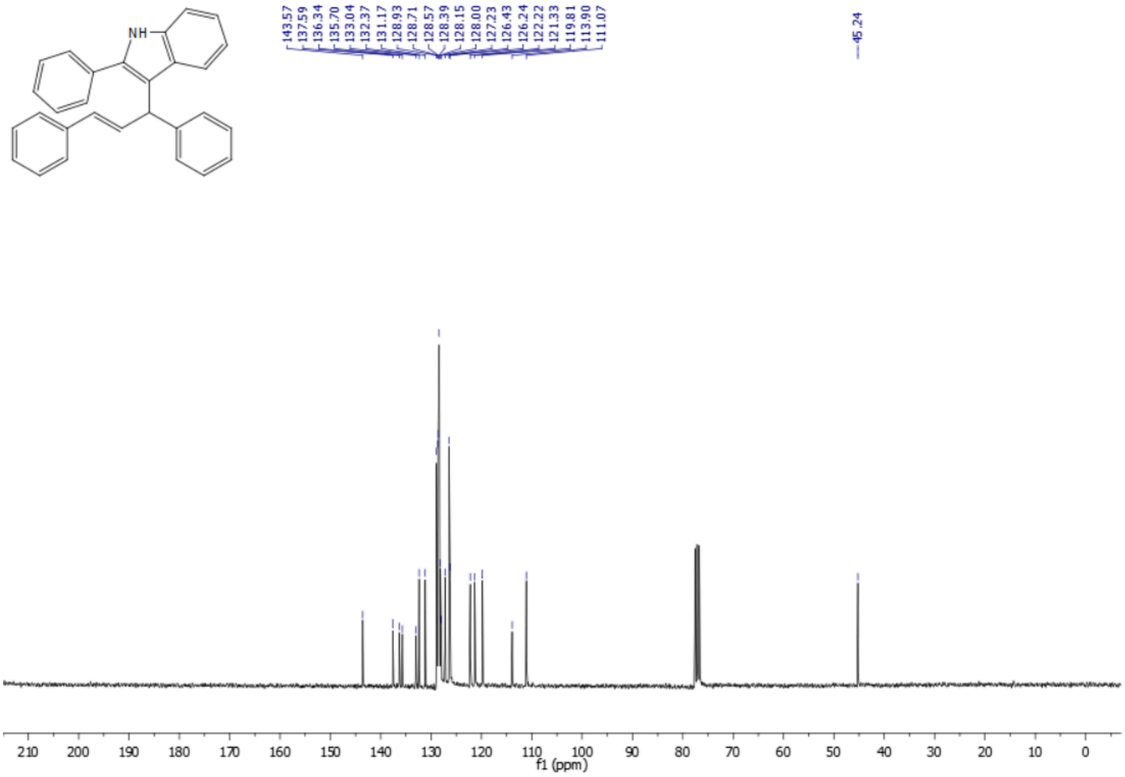


**Figure SI.22.** $^{13}$C NMR spectrum of *(E)*-3-(1,3-diphenylallyl)-2-phenyl-1*H*-indole (**12**)

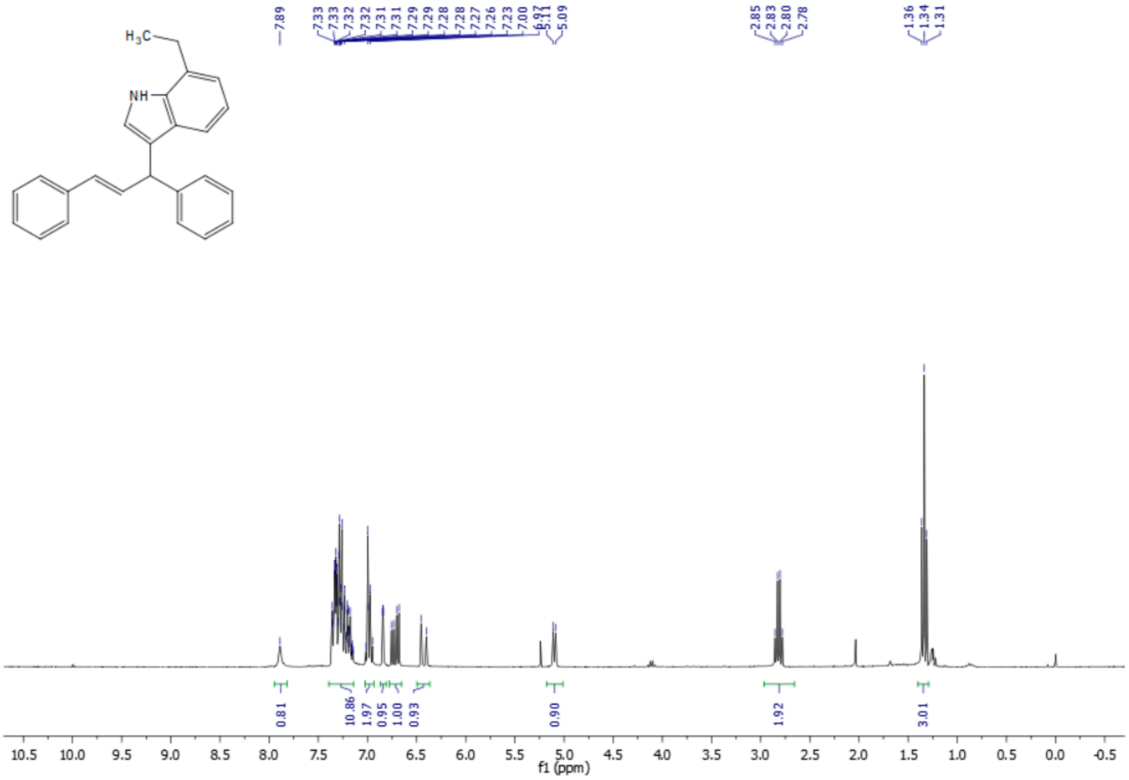

**Figure SI.23.** $^{1}H$ NMR spectrum of *(E)*-3-(1,3-diphenylallyl)-9-ethyl-1*H*-indole (**13**)

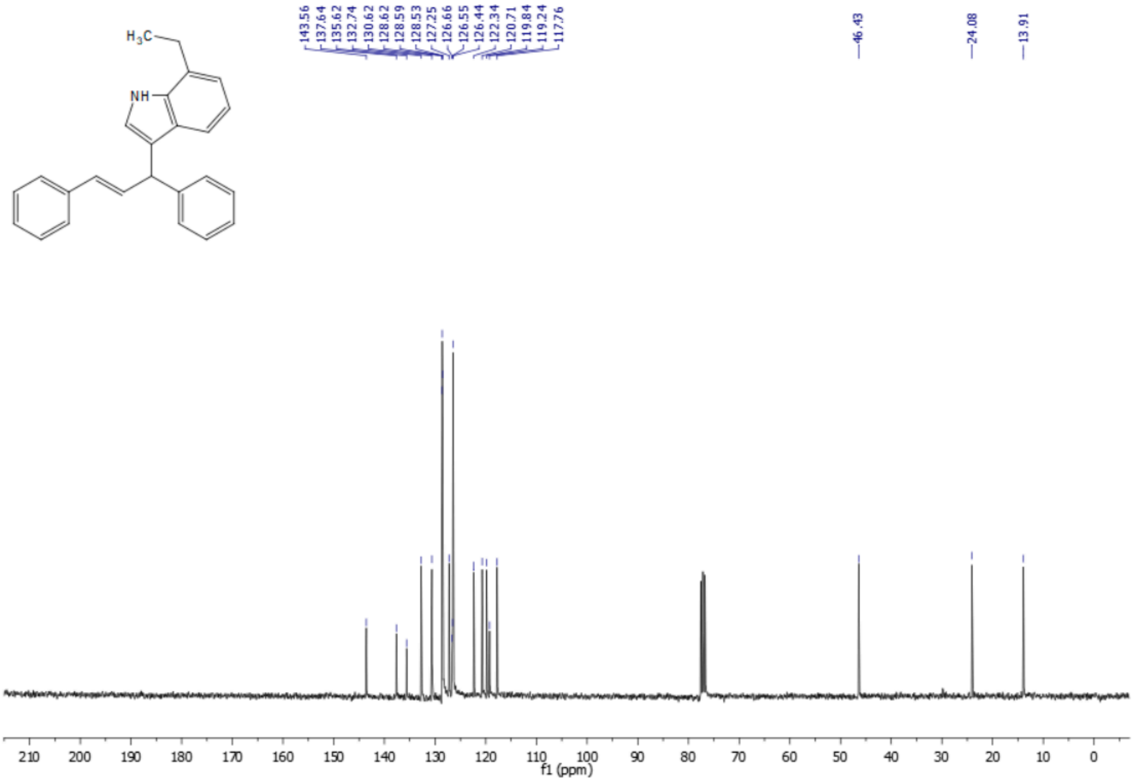

**Figure SI.24** $^{13}C$ NMR spectrum of *(E)*-3-(1,3-diphenylallyl)-9-ethyl-1*H*-indole (**13**)

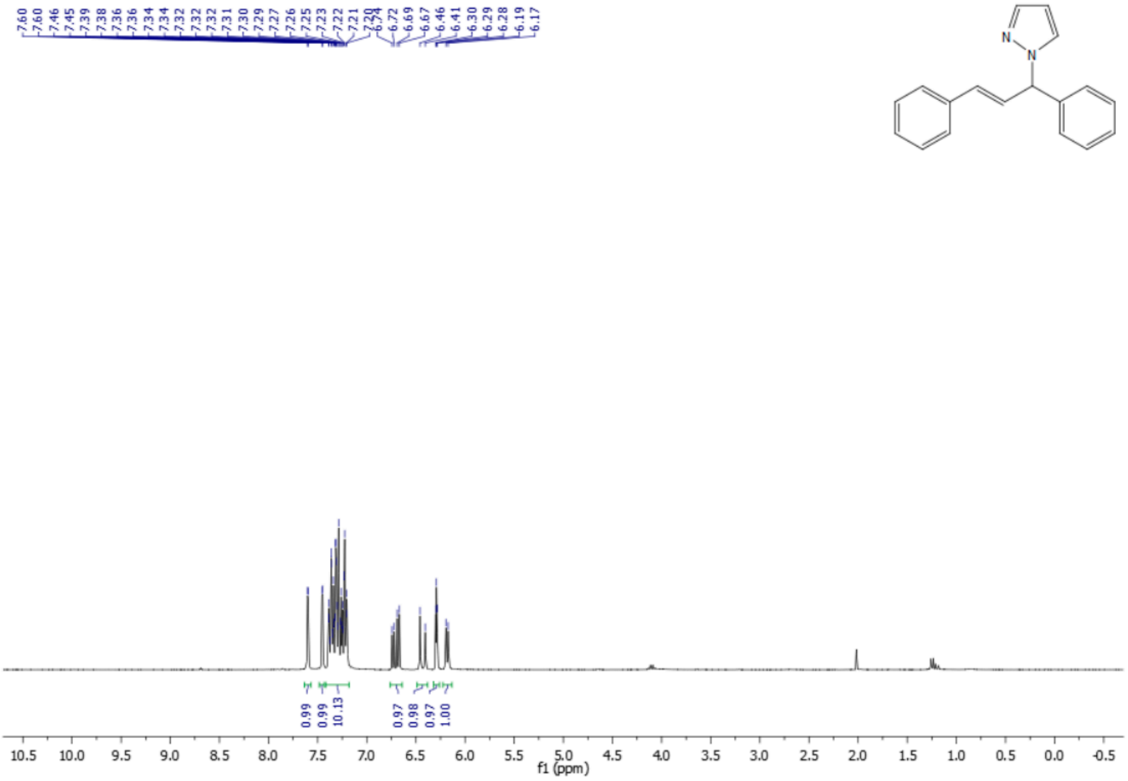


**Figure SI.25.** $^{1}$H NMR spectrum of *(E)*-1-(1,3-diphenylallyl)-1*H*-pyrazole (**14**)

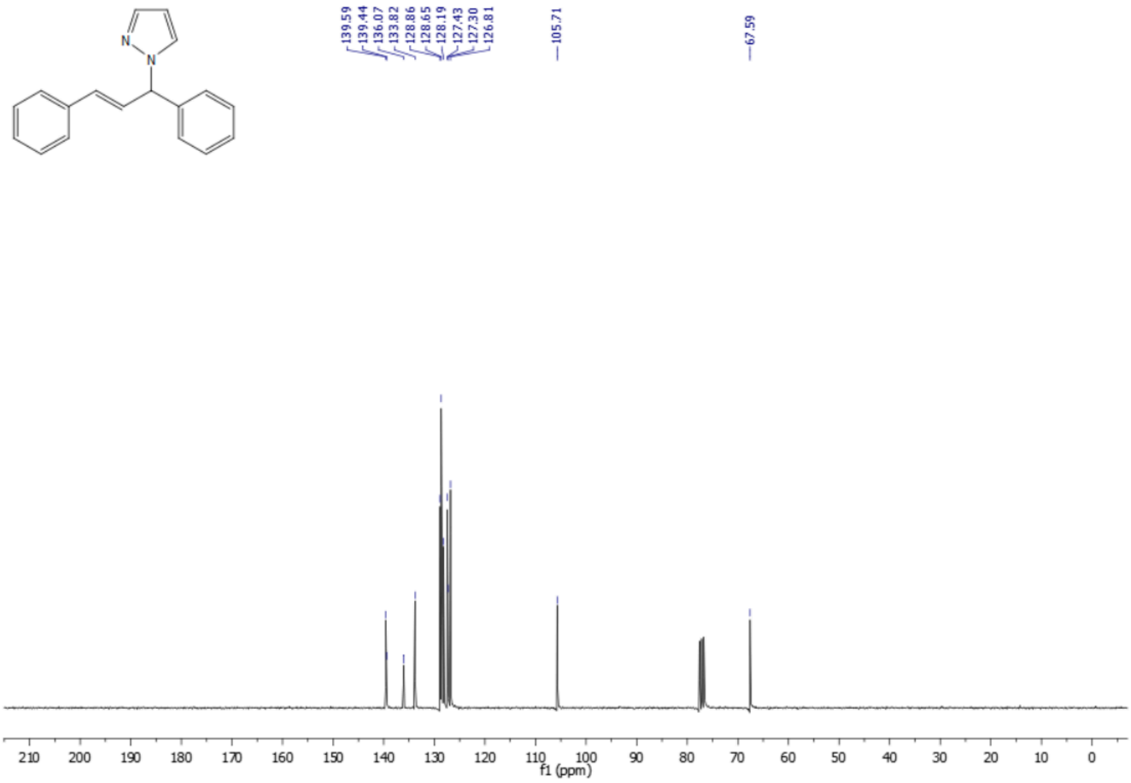


**Figure SI.26.** $^{13}$C NMR spectrum of *(E)*-1-(1,3-diphenylallyl)-1*H*-pyrazole (**14**)

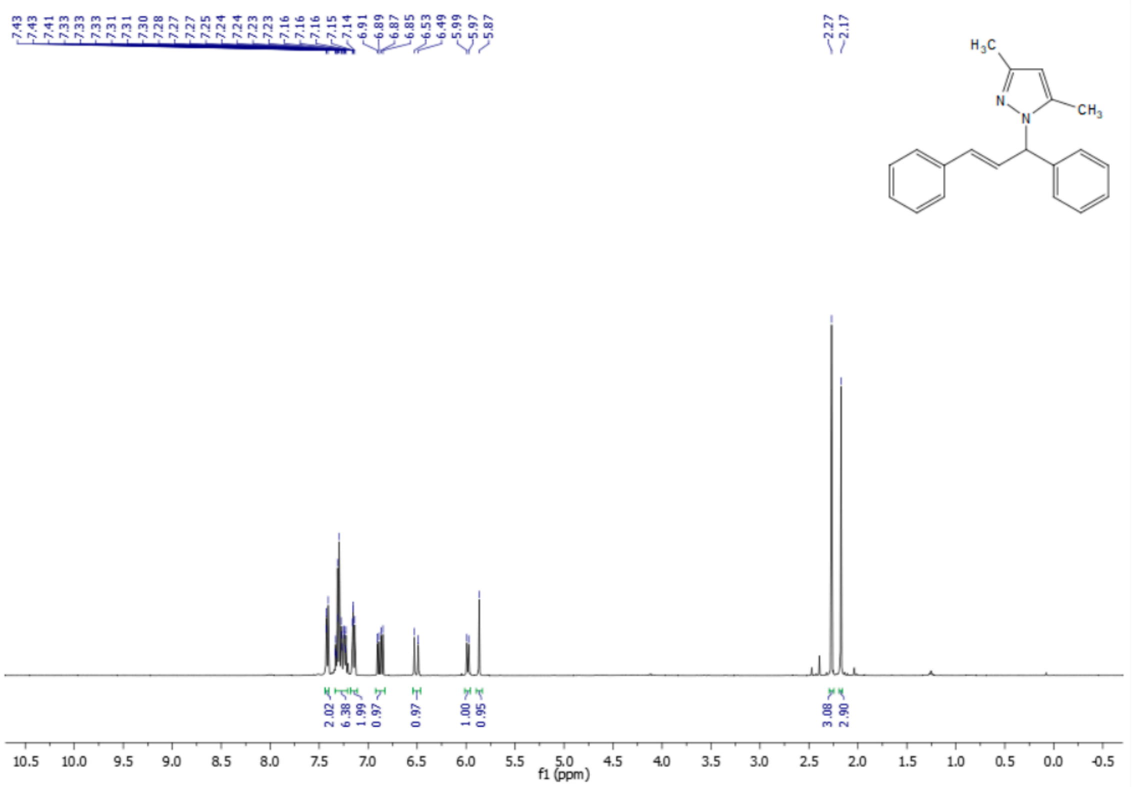

**Figure SI.27.** $^{1}$H NMR spectrum of *(E)*-1-(1,3-diphenylallyl)-3,5-dimethyl-1*H*-pyrazole (**15**)

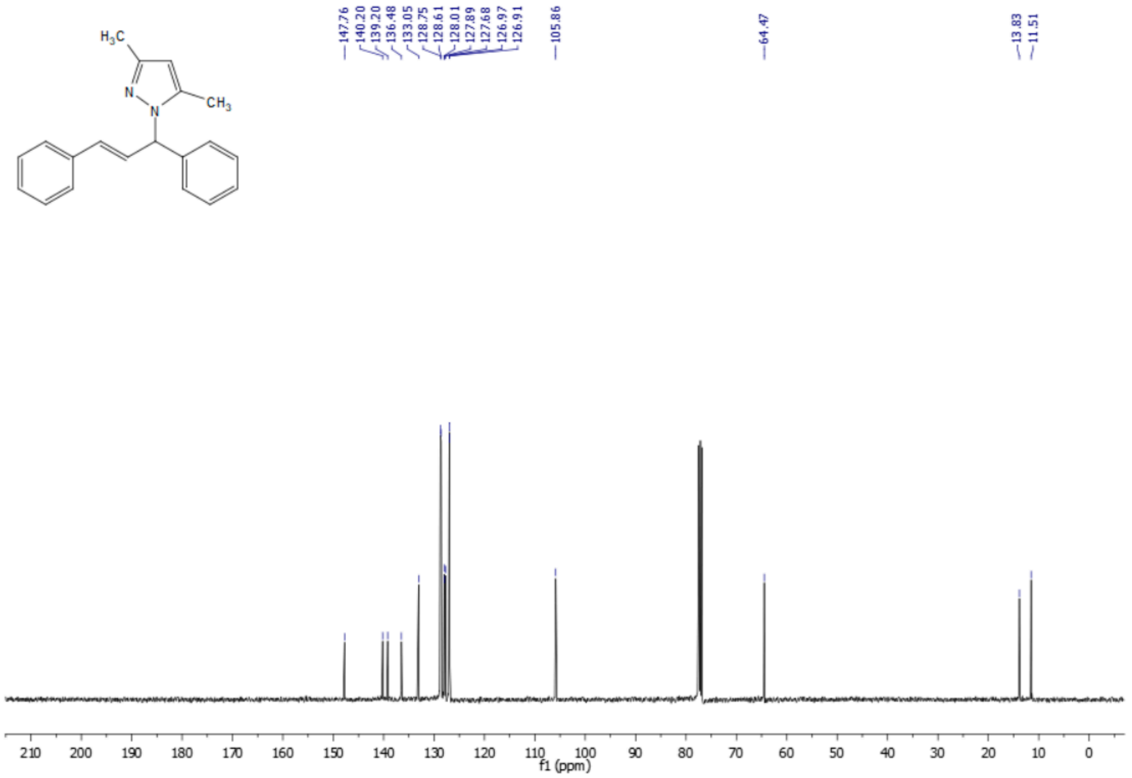

**Figure SI.28.** $^{13}$C NMR spectrum of *(E)*-1-(1,3-diphenylallyl)-3,5-dimethyl-1*H*-pyrazole (**15**)

## References


1. Tanwar, B.; Kumar, A.; Yogeeswari, P.; Sriram, D.; Chakraborti, A.K. Design, development of new synthetic methodology, and biological evaluation of substituted quinolines as new anti-tubercular leads. *Bioorg. Med. Chem. Lett.* **2016**, *26*, 5960–5966. doi:10.1016/j.bmcl.2016.10.082.
2. Gisbert, P.; Albert-Soriano, M.; Pastor, I.M. Effective and sustainable access to quinolines and acridines: A heterogeneous imidazolium salt mediates C-C and C-N bond formation. *Eur. J. Org. Chem.* **2019**, *2019*, 4928–4940. https://doi.org/10.1002/ejoc.201900880.
